# A high-throughput study of visceral organs in CT-scanned pigs

**DOI:** 10.1038/s41598-022-13253-7

**Published:** 2022-06-01

**Authors:** Øyvind Nordbø, Rune Sagevik, Jørgen Kongsro, Kevin Mikkelsen, Arne B. Gjuvsland, Ann-Helen Gaustad, Dan Olsen, Espen W. Remme, Eli Grindflek

**Affiliations:** 1 Norsvin SA, 2317 Hamar, Norway; 2 Geno SA, 2317 Hamar, Norway; 3 The Intervention Centre and Institute for Surgical Research, Oslo University Hospital, Rikshospitalet, Oslo, Norway

**Keywords:** Physiology, Computational science, Scientific data, Cardiovascular biology, Genetics, Animal breeding, Heritable quantitative trait, Medical genetics, Genetics research

## Abstract

It has been debated whether intensive selection for growth and carcass yield in pig breeding programmes can affect the size of internal organs, and thereby reduce the animal’s ability to handle stress and increase the risk of sudden deaths. To explore the respiratory and circulatory system in pigs, a deep learning based computational pipeline was built to extract the size of lungs and hearts from CT-scan images. This pipeline was applied on CT images from 11,000 boar selection candidates acquired during the last decade. Further, heart and lung volumes were analysed genetically and correlated with production traits. Both heart and lung volumes were heritable, with h^2^ estimated to 0.35 and 0.34, respectively, in Landrace, and 0.28 and 0.4 in Duroc. Both volumes were positively correlated with lean meat percentage, and lung volume was negatively genetically correlated with growth (*r*_*g*_ = − 0.48 ± 0.07 for Landrace and *r*_*g*_ = − 0.44 ± 0.07 for Duroc). The main findings suggest that the current pig breeding programs could, as an indirect response to selection, affect the size of hearts- and lungs. The presented methods can be used to monitor the development of internal organs in the future.

## Introduction

Pig breeders have selected pigs for traits like feed efficiency, backfat thickness and daily gain for several decades. It has been debated how this has affected the size of visceral organs^1–5^, and whether the organs have been developing accordingly, to cope with stressors and disease^6^.

Stressors (like e.g. transportation) and diseases are some of the causes of sudden deaths in pigs, and are highly unwanted due to animal welfare concerns, farmers economy, as well as loss of food and thus increased carbon footprint. Sudden death has multifactorial/complex causes^7–9^, relatively low frequency (e.g. 0.15% in U.S market weight pigs)^7^ and, generally, low heritability^10^. In addition, sudden deaths occur more often in commercial farms, which in most cases have large genetic distance to the breeding nucleus. This makes it even more difficult to extract the genetic components and to make any strong genetic progress on survival^11^.

However, since the circulatory and respiratory systems seem to play important roles for sudden deaths, both among finishers^6,12^ and sows^8,13^, one strategy could be to develop indicator traits, e.g. quantifying the capacity of the heart and lungs. Previous studies^2,6^ show that heart lesions and abnormalities, like hypertrophy of the ventricular walls are quite common in finishers and growing pigs, even in animals that seem healthy^6^. More specifically, van Essen et al.^2^ observed that some traits describing the circulatory capacity, like cardiac output and stroke volume in pigs did not scale proportionally with body weight (BW) according to the allometric scaling laws. In addition, they showed that the molecular composition of cardiac tissue was changing towards a less compliant tissue with increased body size, which could impair diastolic filling and predispose for diastolic heart failure.

Increased efficiency of selection, as a result of genomic selection^14^ and increased data quality and quantity^15^, obtained from sensors and associated protocols has facilitated development of broader breeding goals within swine breeding during the last decades. This has led to an inclusion of more health and welfare related traits^16^, like disease tolerance^17^ and survival^18^ in addition to more traditional traits. When selecting animals for growth and carcass yield, it is plausible that also internal organs are affected, as an indirect response to selection, and this could potentially influence the animal’s health. However, large datasets on circulatory and respiratory traits in pigs do not exist.

The first aim of this study is therefore to develop a pipeline for high-throughput analysis of heart and lung size in live pigs. The second aim is to apply this method on a large number of CT-scanned pigs, and to connect the genetics of novel circulatory and respiratory traits to traditional production traits in pig breeding, and to find how the organs have been developing over generations of selection in the studied pig populations.

## Methods

### Animals

5500 Landrace and 5500 Duroc purebred boars in this study were CT scanned as part of the breeding program between 2010 and 2019 as previously described in detail^19^. These boars were born and raised to about 30 kg in different nucleus herds located in Norway, before they were sent to the boar testing station. The boar test includes longitudinal feed and weight recordings, and conformation scoring and CT scanning at the end of the test (at 120 kg)^20,21^.

### Ethics approval

All animals were cared for according to internationally recognized guidelines and laws and regulations for keeping pigs in Norway (Regulation for the keeping of pigs in Norway 2003-02-18-175, 2003; Animal welfare Act 2009-06-19-97, 2009). The data were collected as part of the breeding program in Norsvin.

### Deep learning-based heart and lung measurements

The CT-images of each pig consisted of a stack of approximately 1200 transversal images with 512 × 512 pixels with a resolution of (0.9355 × 0.9355 mm) and with a slice thickness of 1.25 mm. The images covered the entire pig from nose to tail, lying sternally. The pigs were sedated during CT scaning^19^. For segmentation of heart and lungs, a two-step procedure to analyse CT-images of pigs was developed. First, a classification procedure to select images containing heart and lung, followed by a method for segmenting the two organs.

### Classification

A simple convolution neural network (CNN) was trained to classify whether the images contained the chest or not. The network, implemented in TensorFlow^22^, consisted of three convolutional layers using a kernel size of 5 × 5, with 64, 128 and 256 filters respectively. To train this network, a continuous sequence of images (image no. 300–800 from each pig) was extracted first, since the chest region is a continuous subset of these 501 images. All images between the first and last image number in this sequence, containing the lungs, were annotated as chest, while the remaining images were annotated as “not chest”. This was done on 25 pigs, and the training set consisted of 10,521 images (from 21 pigs) while the validation set consisted of 2004 images (from 4 pigs).

The output of this network was the probability for each image to be a part of the chest. To further impose the restriction that the chest is constituted by one continuous sequence of images, we applied a decision tree regressor^23^ which best fitted the raw probability data, using three levels (above chest, chest and below chest). Images having a subsequent probability above 0.5 were then classified as the chest.

### Segmentation

**Figure 1:**
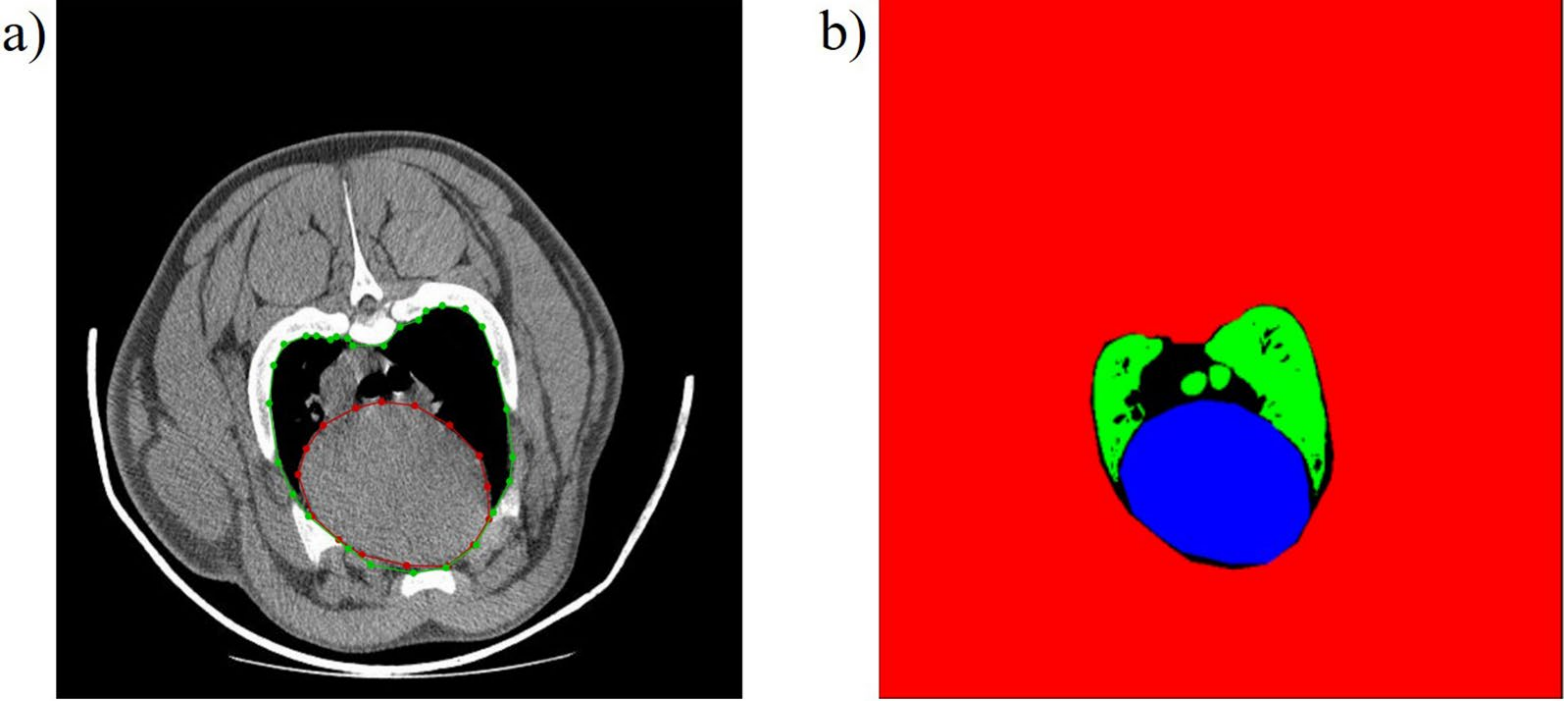
Manual annotation of CT-image. (**a**) Polygons covering the heart only (red) and heart and lung area combined (green). (**b**) Final labelling of heart (in blue), the lungs (in green) and background (in red). The region coloured in black is not assigned to any class.

The sequence of images classified as chest was further analysed by a segmentation CNN. To train the segmentation of lungs and hearts, CT images of DICOM format containing the chest were converted to .png-files and then annotated using LabelMe ^24^. Polygons containing the heart (area surrounded by red line in Fig. 1a) and the region containing both heart and lungs (area surrounded by green line in a) were annotated on 1372 images from 29 pigs (between 38 and 71 images containing the chest from each pig).

The heart region was used directly for training the segmentation CNN, while the mask of the lungs was subsequently automatically segmented by selecting only pixels with Hounsfield unit (HU) below −400^25^ from the mask containing both lungs and heart (see green region in Fig. 1b). The lungs, heart and background mask for these images were then used to train the segmentation CNN. The 1121 images (from 24 pigs) were divided randomly, thus, 80% of images (N = 897) were used for training the network and 20% for validation (N = 224). Data from remaining 5 pigs (N = 251 images) were used as test set for model.

The training data was fitted into a U-net^26^, a simple extension of the CNN presented in^27^, consisting of 6 consecutive contracting and expansive modules, instead of 4. After the last expansive module, a last convolutional layer with a softmax multi-classificator was applied. The model was trained, using dice coefficeient as loss function.

The output of the segmentation network was a pixelwise probability to belong to each of the classes, heart, lung, or background. Pixels having a probability of 0.5 or more to belong to one of the classes, were assigned to their most probable class. Subsequently, to further improve the segmentation of the heart, connected components were used to just include pixels, physically belonging to the largest continuous heart region from the heart class.

### Heart and lung phenotypes

To end up with traits, quantifying the total volume of the heart and the lungs, measured in ml., the total number of pixels containing heart and lungs were calculated across all images of an individual and subsequently multiplied by the pixel size.

The sequence of CT images from 300 to 800 were classified and segmented, using the classification and segmentation CNNs described above. Heart and lung volumes were further investigated to find out how the organ size relates to age and weight at scanning date, using linear regression models (in R^28^) with age and weight as regressors. Further the volumes were analysed to estimate coefficients of the allometric scaling law $y=\alpha {LW}^{\beta }$^29^, by applying linear regression on log-transformed heart and lung volumes (*y)* with a log-transformed live weight (*LW*) at scanning as regressor. A regression coefficient *β* close to one would indicate a proportional relationship between the weight of the animal and the size of the intrathoracic organs.

### Genetic analysis

Further on, heart and lung volumes were included in single- and multi-trait genetic analyses within-breed, using best linear unbiased prediction (BLUP)^30^, using the DMU software^31^. These analyses were conducted using a pedigree that contained all animals with CT-data and five generations of ancestors. Founder animals in these pedigrees were grouped by sex and birth year. Animals born in subsequent birth years were then clustered into genetic groups, so each group contained at least 50 individuals. This resulted in pedigrees containing 11,146 animals and nine genetic groups for Duroc, and 14,184 animals and 17 genetic groups for Landrace. The traits were modelled as

1 $${y}_{ijklmn}={\mathrm{HY}}_{i}+{\mathrm{BM}}_{j}+{\mathrm{PN}}_{k}+\upgamma \times {\mathrm{LW}}_{l}+{\mathrm{p}}_{m}+{\mathrm{a}}_{n}+{\mathrm{e}}_{ijklmn,}$$

with herd year ($\mathrm{HY}$) of the boar’s birth, birth month ($\mathrm{BM}$), and parity number of the dam ($\mathrm{PN}$) as fixed effects. The boar’s phenotype for live weight at scanning date ($\mathrm{LW}$) as a fixed covariate, and the pen (${\mathrm{p}}_{m}$), additive genetic effect of the animal (${\mathrm{a}}_{n}$) and the residual (${\mathrm{e}}_{ijklmn}$) as random effects. First, univariate analyses were performed, and genetic trends were calculated. Secondly, genetic correlations with other relevant traits, taken from the existing breeding programme (shown in Table 1) were estimated. For simplicity, and to avoid convergence issues, the correlations of random effects for pen across traits were set to zero.

**Table 1:** Trait definitions and models for estimation of genetic correlations.

Trait	Definition	Model
Age40	Age at 40 kg's	Equation (1) − $\upgamma \times {\mathrm{LW}}_{l}$
Growth	Average daily gain from 40 to 120 kg's	Equation (1) − $\upgamma \times {\mathrm{LW}}_{l}$
Feed40_120	feed intake 40–120 kg's	Equation (1) − $\upgamma \times {\mathrm{LW}}_{l}$
Yield%	Yield % for carcass (incl. head)	Equation (1)
Lean%	Lean meat % (incl. head)	Equation (1)
Prim%B	Primal % for belly	Equation (1)
Prim%H	Primal % for ham	Equation (1)
Prim%L	Primal % for loin	Equation (1)
Prim%S	Primal % for shoulder	Equation (1)
LoinDepth	Loindepth at 100 kg's	Equation (1) − $\upgamma \times {\mathrm{LW}}_{l}$
BackFat	Backfat at 100 kg's	Equation (1) − $\upgamma \times {\mathrm{LW}}_{l}$

## Results

The segmentation of the lung, heart and background regions had high accuracy in the test-cohort of the 251 images from the five pigs. The overall dice coefficient was 0.992, with higher coefficient for background (0.996) than for lungs (0.950) and heart (0.894).

**Figure 2:**
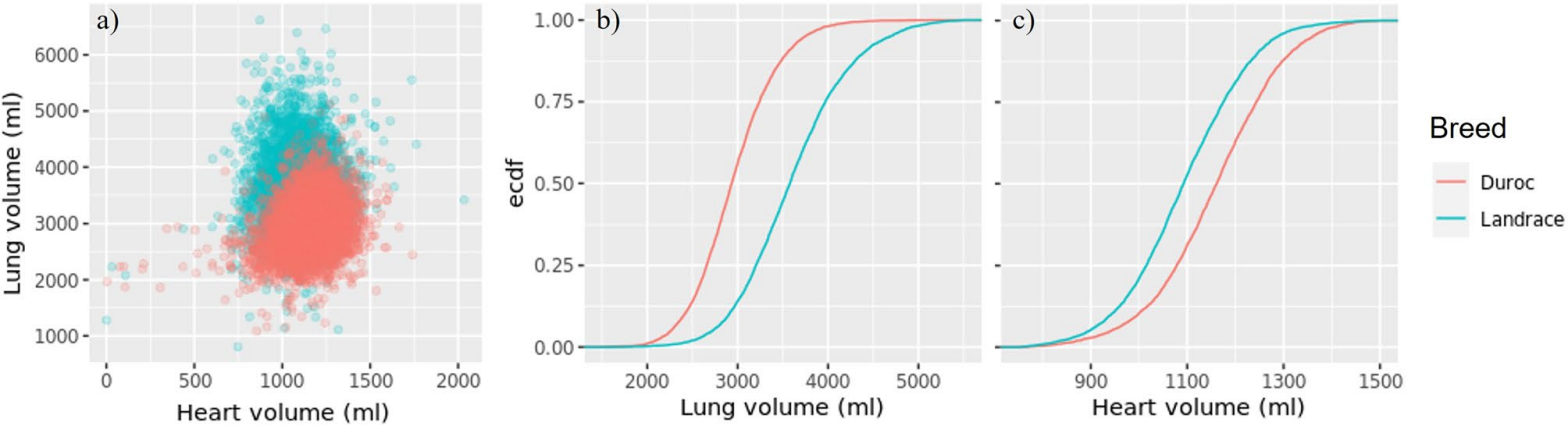
Distribution of heart and lung volumes for Duroc and Landrace pigs predicted from deep learning algorithms as bivariate plot (**a**) and as empirical cumulative distribution functions (ecdf) (in **b,c**).

The distribution of heart and lung volume phenotypes (a), (b) and (c) seem to be close to normally distributed. The average lung volume of Durocs were about 500 ml less than in Landrace, while the average Duroc heart volume was more than 50 ml larger than in Landrace (Fig. 2).

The linear regression models showed that heart volumes were for both lines mainly influenced by body weight of the animal (Table 2).

**Table 2:** Regression coefficients and standard errors for lung and heart volumes for Landrace and Duroc on age and weight at scanning date.

	Weight at scanning (kg)		Age at scanning (days)	
	Reg.coeff	SE	Reg.coeff	SE
**Duroc**				
Lung volume (ml)	0.73	1.75	13.45	0.53
Heart volume (ml)	7.28	0.53	0.01	0.16
**Landrace**				
Lung volume (ml)	8.01	2.33	17.95	0.82
Heart volume (ml)	8.04	0.48	0.54	0.17

For Duroc, the lung volume was mainly determined by the age of the animal, while for Landrace, the lung volume was influenced by both weight and age. Heart volumes were mainly determined by weight for both breeds. Correlations between weight and age at scanning were 0.15 for Duroc and 0.17 for Landrace.

When fitting a linear model on live weight vs. heart and lung volumes (all log-transformed) for Landrace, the $\beta$ values were 0.93 (SE = 0.06) and 0.60 (SE = 0.08) for heart and lung volume, respectively. Corresponding number for Duroc were 0.76 (SE = 0.07) for heart and 0.33 (SE = 0.07) for lung volume.

The heritabilities for heart and lung volumes for Landrace were 0.35 ± 0.04 and 0.34 ± 0.04 while the corresponding numbers for Duroc were 0.28 ± 0.03 and 0.40 ± 0.04. For Landrace, the genetic correlation between the volumes was weak and positive (0.10 ± 0.09), while for Duroc, the correlations were a little stronger (0.34 ± 0.08).

**Figure 3:**
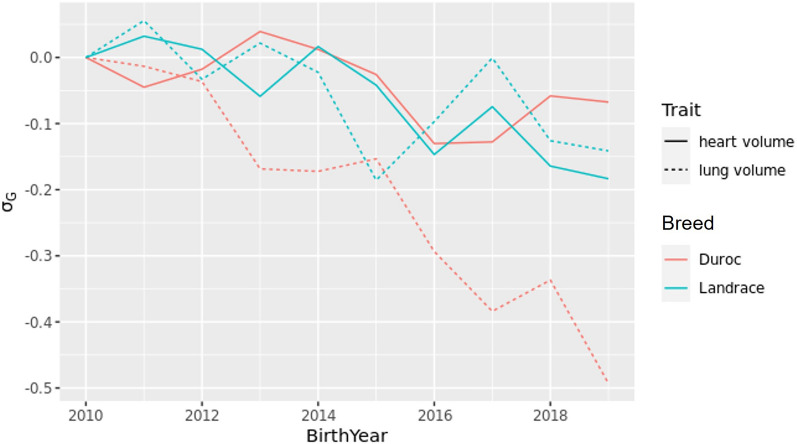
Genetic trends of heart and lung volume, scaled according to the genetic standard deviation, ${\sigma }_{G}$ of the trait.

Genetic trends (average estimated breeding values within birth-year) for lung and heart volumes for Duroc and Landrace are shown in Fig. 3. The genetic trends were scaled according to the genetic standard deviation for the trait and intercept is set to the mean EBV-level of animals born in 2010.

Genetic correlations with other relevant traits for Landrace are shown in Table 3 and corresponding numbers for Duroc are shown in Table 4. Cases where absolute value of genetic correlations are smaller than standard errors are omitted from these tables.

**Table 3:** Genetic correlation between heart and lung volumes and other relevant traits for Landrace.

Trait1	Trait2	Corr	SE
BackFat	HeartSize	−0.28	0.08
LoinDepth	HeartSize	−0.27	0.09
Prim%H	HeartSize	−0.22	0.07
Yield%	HeartSize	−0.14	0.09
Age40	HeartSize	−0.11	0.09
Prim%B	HeartSize	0.12	0.08
Lean%	HeartSize	0.17	0.07
Growth	LungSize	−0.48	0.07
BackFat	LungSize	−0.35	0.08
Prim%B	LungSize	−0.34	0.07
Prim%H	LungSize	0.11	0.07
Yield%	LungSize	0.14	0.08
Prim%S	LungSize	0.22	0.08
Age40	LungSize	0.28	0.09
Lean%	LungSize	0.46	0.06

*BackFat*  backfat at 100 kg's, *LoinDepth*  loindepth at 100 kg's, *Prim%H*  primal % for ham, *Yield%*  yield % for carcass (incl. head), *Age40*  age at 40 kg's, *Prim%B*  primal % for belly, *Lean%*  lean meat % (incl. head), *Growth*  average daily gain from 40 to 120 kg's, *Prim%S* primal % for shoulder.

**Table 4:** Genetic correlation between heart and lung volumes and other relevant traits for Duroc.

Trait1	Trait2	Corr	SE
BackFat	HeartSize	−0.33	0.08
Feed40_120	HeartSize	−0.11	0.08
Growth	HeartSize	−0.11	0.09
Age40	HeartSize	−0.10	0.09
Lean%	HeartSize	0.23	0.07
Growth	LungSize	−0.44	0.07
Prim%B	LungSize	−0.24	0.07
BackFat	LungSize	−0.20	0.08
LoinDepth	LungSize	−0.14	0.08
Feed40_120	LungSize	0.08	0.07
Yield%	LungSize	0.13	0.08
Age40	LungSize	0.17	0.08
Prim%S	LungSize	0.21	0.07
Lean%	LungSize	0.33	0.06

*BackFat*  backfat at 100 kg's, *Feed40_120*  feed intake 40–120 kg's, *Age40* age at 40 kg's, *Growth* average daily gain from 40 to 120 kg's, *Lean%*  lean meat % (incl. head), *Prim%B*  primal % for belly, *LoinDepth*  loindepth at 100 kg's, *Yield%*  yield % for carcass (incl. head), *Prim%S* primal % for shoulder.

## Discussion

In this paper we have developed a deep learning-based pipeline for extraction of novel intratoractic organ phenotypes from CT scanned pigs. This pipeline has been used for a high-throughput analysis of heart and lung size for two purebred pig populations. The heart and lung volumes were moderately heritable (h^2^ 0.28–0.4), which is in the same range as heritabilities estimated for human heart size^32,33^ and human lung volume related phenotypes^34^. Such high heritabilities indicate that the proposed computational pipeline is associated with limited errors, taking the limitations of the current CT acquisition into account (e.g., no ECG-gating, beating heart and respiring animals).

Over the last decade, the genetic trends of visceral organs size in Duroc and Landrace have been negative (Fig. 3). Especially, the lung volumes of Duroc are relatively smaller (half a genetic standard deviation, or 140 ml) at the weight of 120 kg than for ten years ago. This reduction is probably mainly due to its negative genetic correlation to Growth. The Growth seemed to have the strongest genetic correlations to lung size among all tested traits, in both breeds. Phenotypically, the growth rate increased by 15% over the last ten years, causing animals to reach their scanning weight of 120 kg 3 weeks earlier than a decade ago.

Further, the heart and lung sizes seem to be associated with the level of leanness/fatness of the pigs (Tables 3 and 4). For both breeds, the overall lean meat percentage (Lean%) was positively correlated to both heart and lung size. On the other hand, BackFat was negatively correlated with both these traits. The negative genetic correlation between BackFat and heart size is in accordance with the previous findings in pigs^1^ and opposite of observations in human^35^. However, negative correlations between lung volumes and body fat percentage have, like in our study, been found in human^36^.

It has been suggested that such a negative correlation between heart size and BackFat could reflect a pathophysiological change when breeding for leaner pigs^37^. In this study we see, however, that both the lungs and heart volume increase when the BackFat is going down, and to our knowledge, bigger lungs are not associated with any pathophysiological state in pigs. Without claiming any causal pathway, it seems to be a clear correspondence between the size of visceral organs and leanness/fatness. Potentially, this could be explained by a positive relationship between the size of visceral organs, the respiratory/circulatory capacity, the activity, and leanness of the pigs. The potential strength of these correlations should be studied in more detail in the future.

We also observe that the size of the belly (Prim%B) is negatively correlated with lung size. The belly is the most fatty part of the primal cuts, and Prim%B is therefore highly genetically correlated to BackFat^21^.

The lung volumes were on average larger in Landrace than in Duroc, and there might be multiple reasons for that: Landrace have been bred for leanness for decades, and as mentioned above, leanness is highly genetically correlated with lung size (Tables 3 and 4). In addition, since Landrace has been bred for maternal traits like number of teats for many years, it has also increased the number of vertebrae^38^ and thus length of the body. This increased body length might also have affected the space available for lungs. The average heart volumes, on the other hand, are bigger in Duroc than in Landrace.

The animals in this study were all CT-scanned at similar weight (around 120 kg). Hence, the dataset is not optimal to see how organ size scales with body size. When it comes to the volumes of lungs, the results were quite far away from scaling factors suggested by theoretical approaches ($\beta =1)$^29,39^ and cross-species observations^40^. Especially for Duroc ($\beta =0.33)$, but also for Landrace ($\beta =0.60)$, the relationship between body size and lung volume are sublinear. Whether these low scaling factors are due to strong artificial selection^41^, or whether this is because lung volumes are more related to age than weight (Table 2), or a combination of these factors, is not known. A longitudinal study of lung volumes in growing pigs would shed light on these questions.

The allometric scaling factor for heart volumes, $\beta ,$ was equal to 0.76 for Duroc and 0.93 for Landrace pigs. However, the outer volume of the heart is not known to be associated with more relevant traits describing the heart capacity, like e.g., stroke volume or left ventricular diastolic volume. Further, when it comes to allometric scaling laws for outer heart volume, there exists, to our knowledge, not much theory nor measurements. Roughly, the outer volume of the heart is equal to the sum of the volume of the heart tissue plus the heart chamber volumes. The ventricular volumes seem to scale linearly with the body weight^42,43^, but when it comes to the weight of the empty heart, there is some ambiguity in the scientific literature, where both factors of 0.75^2,37^ and 1.0^42,43^ have been reported. Hence, scaling factors for outer heart volume is expected to be either close to one, or between 0.75 and 1.0. The estimated factors here, are at least within this range. It should also be mentioned that the outer heart volume could e.g. potentially be influenced by hypertrophy of ventricular walls and dilatation of ventricular chambers, which is reported to be common in finisher pigs in Canada^6^. Whether such lesions exist among purebred nucleus pigs, is questionable, but compensatory remodelling could potentially give a deviating relationship between heart and body size.

Even if the lung volumes of Durocs have been reduced, probably as an indirect response to selection for e.g. the studied production traits during the last decade, we do not have any indications that this has implications for the animal’s health. Duroc is the most common terminal sire line used in Norway, and the finisher survival in Norway has improved the last years^44^. By studying genetic correlations between lung and heart size and survival/longevity, preferably with data from commercial environments, we could find out what is the preferrable direction of genetic progress of internal organs to improve the robustness. This pipeline is therefore a useful tool to monitor the progress of development of internal organs of live animals in a sustainable breeding program.

To improve the interpretation of the results, a refined deep learning model for measuring the lung volume would be beneficial. In the current study, only the volume of air inside lungs was investigated. Differentiation between anatomical changes in the form of a smaller chest, or connective tissue and pathological conditions could be done by assigning the pixels within the manually segmented chest area (green line in Fig. 2a) that do not belong to heart or lungs, into an own class (represented by the black regions in Fig. 2b). This class would represent the amount of connective tissue, blood vessels, or any pathological condition. Summing this class with heart and lung volumes would constitute the whole intrathoracic volume. Another future improvement would be to develop new CT-protocols, with e.g., ECG-gating or ability to simultaneously measure respiratory phase. This would further increase the potential to extract even more relevant cardiac and respiratory traits in live pigs. Inclusion of other internal organs, like liver, kidneys etc. into this pipeline would also be a potential extension of this work.

## Conclusion

In this study, we developed a deep learning-based computational pipeline for extracting characteristics of the visceral organs in live CT-scanned pigs. This pipeline was applied to 11.000 scanned pigs, and the genetic parameter analysis for heart and lung volumes were performed. These traits were moderately heritable (h^2^ 0.28–0.4), and both showed a slightly negative genetic trend over the last decade. Especially the lung size of the Duroc has been reduced, and this could probably be explained by the relatively strong negative genetic correlation to growth. As such, this work provided useful knowledge on how visceral organs develop over time together with intensive breeding for production, and a very useful tool to ensure the biological balanced composition of the animal in a sustainable breeding program.

## Data Availability

The data that support the findings of this study are available from Norsvin SA, but restrictions apply to the availability of these data, which were used under license for the current study, and thus are not publicly available. However, data is available from the authors upon reasonable request and with permission of Norsvin SA.

## References

[1] Cliplef RL, McKay RM (1993). Visceral organ weights of swine selected for reduced backfat thickness and increased growth rate. Can. J. Anim. Sci..

[2] van Essen GJ (2018). Cardiovascular function of modern pigs does not comply with allometric scaling laws. Sci. Rep..

[3] Tereszkiewicz K, Molenda P, Pokrywka K (2011). Influence of selected factors on the weight of internal organs of fatteners. Acta Sci. Pol. Zootech..

[4] van Essen GJ (2011). Cardiovascular performance of adult breeding sows fails to obey allometric scaling laws1. J. Anim. Sci..

[5] van Essen GJ (2009). Does cardiovascular performance of modern fattening pigs obey allometric scaling laws?. J. Anim. Sci..

[6] Zurbrigg K (2019). Rapid communication: A comparison of cardiac lesions and heart weights from market pigs that did and did not die during transport to one Ontario abattoir. Transl. Anim. Sci..

[7] Ritter MJ, Yoder CL, Jones CL, Carr SN, Calvo-Lorenzo MS (2020). Transport losses in market weight pigs: II. U.S. incidence and economic impact. Transl. Anim. Sci..

[8] Segura-Correa JC, Ek-Mex E, Alzina-López A, Segura-Correa VM (2011). Frequency of removal reasons of sows in Southeastern Mexico. Trop. Anim. Health Prod..

[9] Zurbrigg K (2017). Pig-level risk factors for in-transit losses in swine: A review. Can. J. Anim. Sci..

[10] Cheng J (2020). Genetic analysis of disease resilience in wean-to-finish pigs from a natural disease challenge model. J. Anim. Sci..

[11] Iversen MW, Nordbø Ø, Gjerlaug-Enger E, Grindflek E, Meuwissen THE (2020). Predicting survival and longevity of sows using purebred and crossbred data1. Transl. Anim. Sci..

[12] Piva MM (2020). Causes of death in growing-finishing pigs in two technified farms in southern Brazil. Pesqui. Veterinária Bras..

[13] Drolet R, D’Allaire S, Chagnon M (1992). Some observations on cardiac failure in sows. Can. Vet. J..

[14] Meuwissen THE, Hayes BJ, Goddard ME (2001). Prediction of total genetic value using genome-wide dense marker maps. Genetics.

[15] Piñeiro C (2019). Big (pig) data and the internet of the swine things: a new paradigm in the industry. Anim. Front..

[16] Hermesch, S. Breeding for improved welfare of growing pigs. in *Breeding Focus 2016-Improving Welfare.* 77–88. (2016).

[17] Knap PW, Doeschl-Wilson A (2020). Why breed disease-resilient livestock, and how?. Genet. Sel. Evol..

[18] Merks JWM, Mathur PK, Knol EF (2012). New phenotypes for new breeding goals in pigs. Animal.

[19] Aasmundstad T, Kongsro J, Wetten M, Dolvik NI, Vangen O (2013). Osteochondrosis in pigs diagnosed with computed tomography: Heritabilities and genetic correlations to weight gain in specific age intervals. Anim. Int. J. Anim. Biosci..

[20] Gjerlaug-Enger E, Kongsro J, Odegård J, Aass L, Vangen O (2012). Genetic parameters between slaughter pig efficiency and growth rate of different body tissues estimated by computed tomography in live boars of Landrace and Duroc. Anim. Int. J. Anim. Biosci..

[21] Kongsro J, Gangsei LE, Karlsson-Drangsholt TM, Grindflek E (2017). Genetic parameters of in vivo primal cuts and body composition (PigAtlas) in pigs measured by computed tomography (CT). Transl. Anim. Sci..

[22] Abadi, M. *et al.**TensorFlow: Large-Scale Machine Learning on Heterogeneous Distributed Systems*. arXiv160304467 Cs. (2016).

[23] Pedregosa F (2011). Scikit-learn: Machine learning in Python. J. Mach. Learn. Res..

[24] Wada, K. *labelme: Image Polygonal Annotation with Python*. 10.5281/zenodo.5711226 (2016).

[25] Elsayed, O., Mahar, K., Kholief, M. & Khater, H. A. Automatic detection of the pulmonary nodules from CT images. in *2015 SAI Intelligent Systems Conference (IntelliSys)*. 742–746. 10.1109/IntelliSys.2015.7361223 (2015).

[26] Ronneberger, O., Fischer, P. & Brox, T. U-Net: Convolutional networks for biomedical image segmentation. in *Medical Image Computing and Computer-Assisted Intervention—MICCAI 2015* (eds. Navab, N., Hornegger, J., Wells, W. M. & Frangi, A. F.). 234–241. 10.1007/978-3-319-24574-4_28 (Springer, 2015).

[27] Lamba, H. Understanding semantic segmentation with UNET. in *Medium*. https://towardsdatascience.com/understanding-semantic-segmentation-with-unet-6be4f42d4b47 (2019).

[28] R Core Team. *R: A Language and Environment for Statistical Computing*. (R Foundation for Statistical Computing, 2020).

[29] West GB, Brown JH, Enquist BJ (1997). A general model for the origin of allometric scaling laws in biology. Science.

[30] Henderson CR (1975). Best linear unbiased estimation and prediction under a selection model. Biometrics.

[31] Madsen, P. & Jensen, J*. A User’s Guide to DMU. A Package for Analysing Multivariate Mixed Models. Version 6, Release 5.2*. http://dmu.agrsci.dk/DMU/Doc/Current/dmuv6_guide.5.2.pdf (2013).

[32] Bella JN (2004). Heritability of left ventricular dimensions and mass in American Indians: The Strong Heart Study. J. Hypertens..

[33] Jin Y (2011). Heritability of left ventricular structure and function in Caucasian families. Eur. J. Echocardiogr..

[34] Chen Y (1999). Genetic epidemiology of pulmonary function. Thorax.

[35] Hense H-W (1998). The associations of body size and body composition with left ventricular mass: Impacts for indexation in adults. J. Am. Coll. Cardiol..

[36] Sutherland TJT, McLachlan CR, Sears MR, Poulton R, Hancox RJ (2016). The relationship between body fat and respiratory function in young adults. Eur. Respir. J..

[37] Yang TS, Lin JH (1997). Variation of heart size and its correlation with growth performance and vascular space in domestic pigs. Anim. Sci..

[38] van Son M (2019). A QTL for number of teats shows breed specific effects on number of vertebrae in pigs: Bridging the gap between molecular and quantitative genetics. Front. Genet..

[39] Noël, F., Karamaoun, C. & Mauroy, B. *The Origin of the Allometric Scaling of Lung’s Ventilation in Mammals*. hal-02567829v2 (2020).

[40] Stahl WR (1967). Scaling of respiratory variables in mammals. J. Appl. Physiol..

[41] Bolstad GH (2015). Complex constraints on allometry revealed by artificial selection on the wing of *Drosophila melanogaster*. Proc. Natl. Acad. Sci..

[42] Dawson TH (2014). Allometric relations and scaling laws for the cardiovascular system of mammals. Systems.

[43] Holt JP, Rhode EA, Kines H (1968). Ventricular volumes and body weight in mammals. Am. J. Physiol..

[44] Animalia. *Ingris**, **Årsstatistikk 2020*. https://www.animalia.no/no/Dyr/husdyrkontrollene/ingris/arsstatistikk/.

